# Vertical Distribution and Drivers of Antibiotic Resistance Genes in Agricultural Soil Irrigated with Livestock Wastewater

**DOI:** 10.3390/microorganisms13030610

**Published:** 2025-03-06

**Authors:** Ming Shang, Yongchao Gao, Liwen Zheng, Lei Ji, Jianhua Du, Xue Kong, Hui Wang, Feng Shi, Hailun Wang, Jianhui Liu, Xiaodong Yang, Zeyu Wang

**Affiliations:** 1 Shandong Provincial Key Laboratory of Applied Microbiology, Ecology Institute, Qilu University of Technology (Shandong Academy of Sciences), Jinan 250103, China; sm17667939671@163.com (M.S.); zhenglw@qlu.edu.cn (L.Z.); jilei.1010@163.com (L.J.); kongx@qlu.edu.cn (X.K.); shifeng1224cn@126.com (F.S.); wanghl0215@163.com (H.W.); 15665891128@163.com (J.L.); wmicro421@outlook.com (Z.W.); 2 WSP Australia Pty Limited, Level 3, Mia Yellagonga Tower 2, 5 Spring Street, Perth, WA 6000, Australia; jason.du@wsp.com; 3 School of Resources and Environment, University of Jinan, Jinan 250022, China; hwang_118@163.com; 4 Department of Geography & Spatial Information Technology, Ningbo University, Ningbo 315211, China; yangxiaodong@nbu.edu.cn

**Keywords:** antibiotic resistance genes, antibiotic resistance bacteria, livestock wastewater, agricultural soil

## Abstract

Livestock wastewater reuse could be a potential source for the distribution of antibiotics, antibiotic resistance bacteria (ARB), and antibiotic resistance genes (ARGs) in agricultural soil. In this study, soil samples were collected from different depths (0–60 cm) of farmland that has been subjected to long-term application of livestock wastewater. The vertical distribution of antibiotics, bacterial communities, and ARGs were assessed to identify the driving factors that could potentially influence the distribution of ARB and ARGs. The results demonstrated distinguished distributions of antibiotics along the soil depths, with tetracyclines (TCs) mainly found in the top 10 cm of the soil (0.11–0.31 μg/kg), while quinolones (QNs), sulfonamides (SAs), and macrolides (MLs) were detected in all 60 cm of soil depth (0.01–0.22 μg/kg). The selection pressure of antibiotics to microorganisms led to the proliferation of ARB, especially tetracycline-resistant bacteria and erythromycin-resistant bacteria. In terms of the distribution/abundance of ARGs, *novA* and *tetA* (58) were relatively higher in 0–10 cm surface soil, while *vanRM* and *vanRF* were mainly detected in the deeper soil. Different ARGs may have the same host bacteria, which lead to the emergence of multidrug resistant bacteria, such as *Ilumatobacter* sp., *Aggregatilinea* sp., *Rhabdothermincola* sp., and *Ornithinimicrobium* sp. Soil pH, electrical conductivity (EC), and moisture content (MC) could affect the distribution and proliferation of ARB and were found negatively correlated with most of the ARGs except *macB*. Therefore, it is potentially possible to eliminate/inhibit the spread of ARGs by adjusting these soil parameters. These findings provide insights into the distribution and dissemination of antibiotics, ARB, and ARGs in agricultural practices of livestock wastewater irrigation and provide effective mitigation strategies to ensure the safe use of livestock wastewater in agriculture.

## 1. Introduction

Antibiotic resistance is considered one of the most prominent global health challenges of the 21st century, posing a serious threat to ecosystem and human health [1]. Long-term extensive use of various antibiotics in medicine, agriculture, and livestock farming is found to be linked with the prevalence of antibiotic resistance bacteria (ARB) and antibiotic resistance genes (ARGs) in the environment [2,3,4]. The overuse of veterinary antibiotics in intensive livestock farming is particularly problematic [5]. Antibiotics are hardly fully absorbed by animals, and it is found that more than half of the antibiotic intake was excreted through feces and urine in their original forms or related metabolites [6]. Manure and livestock wastewater can provide nutrients and organic matter to boost plant growth and improve soil quality and are widely used as fertilizer in parts of China. However, the livestock waste could represent a potential source and cause of the spread of antibiotics, ARB, and ARGs in the agricultural land [7].

Tetracyclines (TCs), macrolides (MLs), and sulfonamides (SAs) are the most accumulated antibiotics in soils, with concentrations ranging from 0.02 µg/kg to 7000 µg/kg [8]. The vertical distribution of the antibiotics in soil is considered to be related to the structure and physiochemical properties of the antibiotics [9]. For example, the benzene rings and lipid rings in TCs are more likely to form complexes with the organic matter in soil, therefore, the concentrations of TCs are relatively higher in the topsoil in comparison to the bottom soil, which generally has lower organic matter content. Tang et al. [10] concluded that the long-term application of manure generally led to the increase in TC content of rice soils, and the concentration of TCs decreased with the increase in soil depth. On the contrary, SAs are mainly found in deep soil layers due to the weak adsorption ability of aniline and amide groups with soil and higher leachability [11]. Wei et al. [12] investigated the presence of 13 veterinary drugs in animal manure fertilized soils in East China, and found that the concentrations of three SAs and two fluoroquinolones (FQs) were higher in soil depths of 20–40 cm and 40–60 cm in comparison to 0–20 cm. More seriously, antibiotics were detected in groundwater [13]. Kivits et al. [14] investigated antibiotics in groundwater with a depth of 23 m in two livestock-intensive areas of the Netherlands, the concentrations of sulfamethazine (SM2), sulfamethoxazole (SMZ), lincomycin (LIN), chloramphenicol (CAP), ciprofloxacin (CIP), and sulfadiazine (SD) ranged from 0.3 to 18 ng L^−1^. The residual antibiotics contributed to the overall selective pressure to microorganisms and accelerated the development of antibiotic resistances [15]. Peng et al. [16] conducted a comprehensive survey on the ARGs pollution characteristics of six major livestock and poultry manures in Ningxia, China. A total of 329 ARGs were detected, and the highly abundant ARGs were predominantly positively correlated with the antibiotics used in livestock breeding.

Vertical gene transfer (VGT) and horizontal gene transfer (HGT) are the two main pathways by which ARGs are spread. VGT increases the abundance of ARGs with the proliferation of host bacteria. HGT increases the diversity, host range, and abundance of ARGs [7,17]. HGT, which will lead to cross-species transmission, has attracted more attention in recent years. There is research on the bioaccumulation and biomagnification effects of veterinary antibiotics and ARGs in vegetables and crops [18,19,20,21,22]. ARGs could therefore enter the human body through ingestion and colonize in the human gut. Therefore, it is important to understand the distribution, migration rules, and affecting factors of antibiotics, ARB, and ARGs in soil [23].

In this study, soil samples were collected from different depths from farmland subject to long-term application of treated livestock wastewater. The physicochemical properties of the soil samples and the speciation of the main antibiotics, bacterial communities, and the abundance of the ARGs were analyzed. The objectives were to investigate the distribution profile of the antibiotics, ARGs, and ARB and assess the factors that could influence the distribution, with the aim of identifying the prevention and control measures of antibiotic resistance in agricultural land.

## 2. Materials and Methods

### 2.1. Study Area and Sample Collection

The study area is located on a dairy farm (34°84′ N, 115°58′ E) of Shandong Province, China. Based on the Chinese Soil Classification System, the soil type is fluvo-aquic soil. Three sample locations (labeled as CL) were selected from the farmland near the dairy farm and have been irrigated with treated livestock water for more than 10 years. The treatment process of the livestock wastewater mainly focuses on the removal of nitrogen and phosphorus and does not take specific measures to remove antibiotics, ARB, and ARGs [24]. Another three sample locations were also selected in the second block of farmland, separated by road and drainage trenches and 10 m away from CL, which was not irrigated with the treated livestock wastewater, was selected as the control (Control, CK). There were six sampling locations altogether. In each sampling location, soil samples were collected at different layers using a quartering method and homogenized to form a representative composite sample. The sampling layers were 0–5 cm, 5–10 cm, 10–20 cm, 20–40 cm, and 40–60 cm and were numbered from 1 to 5 with increasing depths (CL1-CL5, CK1-CK5). A total of 30 soil samples were collected. The collected soil samples were dispensed into 10 mL sterile centrifuge tubes and sent to a commercial laboratory (Majorbio Bio-Pharm Technology Co., Ltd., Shanghai, China) for high-throughput metagenomic sequencing using a foam box with dry ice. The other samples were transported back to the laboratory (Institute of Ecology, Shandong Academy of Sciences, Jinan, China) at 4 °C and preprocessed within 12 h. Fresh samples were stored at −80 °C for the detection of antibiotics and ARB and saved not more than 30 days before analysis. The other part of the samples was air-dried, ground, and passed through a 2 mm sieve for the determination of the soil’s physical and chemical properties.

### 2.2. Chemical Properties of the Samples

Soil pH was measured by a pH probe (HACH-PHC101, America) at a soil-to-water ratio of 1:5 (*w*/*v*). Soil electrical conductivity (EC) was measured by an EC probe (HACH-CDC40101, America) at a soil-to-water ratio of 1:5 (*w*/*v*). In detail, the 5.0 g soil sample was weighed and placed in a 50 mL centrifuge tube, and 25 mL double distilled water was added. After sealing the container, it was oscillated violently with a horizontal oscillator for 2 min. After standing for 30 min, the determination of pH and EC was completed within 1 h. Moisture content (MC) of the soil was determined by the drying method [25].

### 2.3. Analysis of the Antibiotics in Soil

The antibiotics in the soil samples were extracted by ultrasonic extraction followed by solid phase extraction (SPE) [26,27]. Briefly, 5.0 g of soil samples were extracted with Na_2_EDTA and a mixture of phosphate buffer and acetonitrile (1:1, *v*/*v*). The extraction was repeated three times with ultrasound and solid phase extraction using a hydrophilic–lipophilic balance (HLB) column. Then, the extract was analyzed using ultra-high performance liquid chromatography (UHPLC) coupled with a mass spectrometry (MS) system (vanqushTSQ Endura; Thermo Scientific, Waltham, MA, USA). The mobile phase A was 0.1% formic acid in water, and the mobile phase B was 0.1% formic acid-methanol/acetonitrile (*v*/*v* = 1:1). The detailed mass spectrometry parameters, elution gradients, and ion source conditions were provided in the Appendix A (Table A1 and Table A2).

### 2.4. ARB Colony Counting

Soil suspension was prepared by mixing 1.0 g of previously frozen fresh soil sample and 9 mL of sterile water. The soil suspension was then diluted in gradient, and 100 μL was taken to coat on different LB solid medium plates containing tetracycline (TC) (16 μg/mL), oxytetracycline (OTC) (16 μg/mL), CIP (1 μg/mL), norfloxacin (NOR) (8 μg/mL), erythromycin (ERY) (8 μg/mL), and polymyxin B sulphate (PMB) (4 μg/mL). The ARB colonies were counted after being incubated for 48 h at 37 °C.

### 2.5. Metagenomic Sequencing and Bioinformatics Analysis

#### 2.5.1. Library Preparation and Sequencing

Metagenomic sequencing was performed to analyze the composition of the bacterial community and the relative abundance of ARGs in the samples using the Illumina NovaSeq platform at Majorbio Bio-Pharm Technology Co., Ltd. (Shanghai, China). Total genomic DNA was extracted from 0.5 g soil samples using the Magnetic Soil and Stool DNA Kit (DP712, TIANGEN, Beijing, China), repeat 3 times for each plot. The DNA extraction process strictly followed the standard operating procedures, and a blank control was set up to exclude possible contamination. Concentration and purity of the extracted DNA were determined using TBS-380 and NanoDrop2000, respectively. Library construction was accomplished using the NEXTFLEX^®^ Rapid DNA-Seq Kit (Bioo Scientific, Austin, TX, USA). The qualified libraries were sequenced using the Illumina NovaSeq 6000 platform (Illumina Inc., San Diego, CA, USA), generating approximately 12 Gb of metagenomic data per DNA sample. Raw sequences from this study have been submitted to the National Center for Biotechnology Information (NCBI) Sequence Read Archive (SRA) under accession number PRJNA1200920.

#### 2.5.2. Analysis of Sequencing Information

A certain percentage of low-quality data may exist in the raw data obtained from sequencing. To ensure the accuracy and reliability of subsequent information analysis results, fastp (https://github.com/OpenGene/fastp, version 0.20.0, accessed on 5 August 2024) was used to perform quality control of the raw data obtained and to obtain the clean data for subsequent analysis. The R package decontam (version 1.8.0) was used to detect and remove the contaminated sequences automatically. Data quality control retained high-quality paired-end reads and single-end reads by removing reads that were less than 50 bp in length after shearing, had an average base quality value of less than 20, and contained N bases. The optimized sequences were spliced and assembled using MEGAHIT (https://github.com/voutcn/megahit, version 1.1.2, accessed on 5 August 2024). The spliced sequences with ≥ 300 bp contigs were selected as the final assembly results. Contigs ≥ 300 bp in the splicing result will be selected as the final assembly result. The contigs in the splicing results were analyzed using Prodigal/MetaGene (https://metagene.nig.ac.jp/metagene/metagene.html, accessed on 5 August 2024). Open Reading Frames (ORFs) were predicted for contigs in the splicing results. Genes with nucleic acid lengths greater than or equal to 100 bp were selected and flipped and translated into amino acid sequences.

### 2.6. Statistical Analyses

Gene abundance is expressed as the number of reads contained in the gene as a percentage of all reads in the sample:

(1) $${Gene}_{i}=\frac{R_{i}}{\sum_{1}^{n}\left(R_{j}\right)}$$

where R_i_ represents the number of reads compared to Gene_i_ in the sample; $\sum_{1}^{n}\left(R_{j}\right)$ represents the sum of reads corresponding to all genes in that sample.

Mean and standard error of the data were calculated using Microsoft Excel 2007 (Microsoft Corporation, Redmond, WA, USA). The normality of the data was evaluated by a Shapiro–Wilk test, and an analysis of variance (ANOVA) was carried out using SPSS (version 25.0). Correlation analysis was carried out using Origin Pro. 2024 (OriginLab Corporation, Northampton, MA, USA). Correlation heatmap plots, principal coordinate analysis (PCoA) based on the Bray–Curtis distance, analysis of similarities (ANOSIM), and non-metric multidimensional scaling (NMDS) were made using the “vegan” package (version 2.4.3) of R (V. 4.3.0). Linear discriminant analysis (LDA) was performed using LEfSe (http://galaxy.biobakery.org/) to identify species or functions that significantly differed in samples.

## 3. Results

### 3.1. Distribution of Antibiotics in the Investigated Farmland

**Figure 1:**
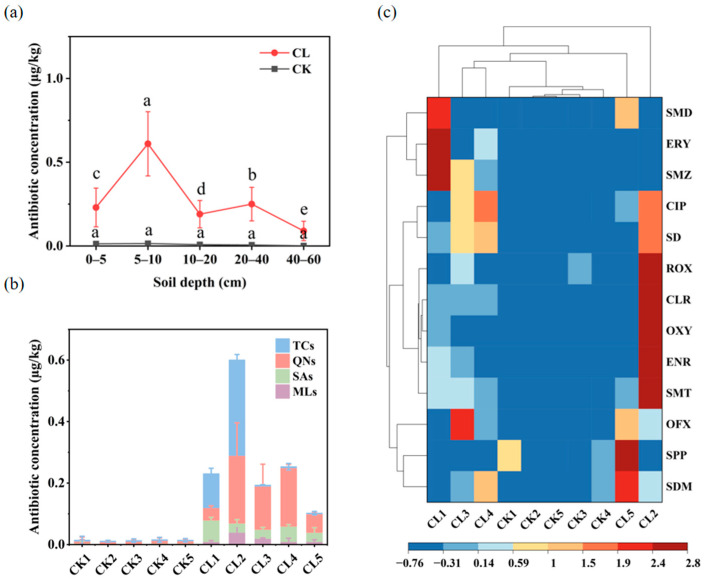
The distribution profile of antibiotics in soil under different soil depths. (**a**) The concentration of total antibiotics. Error bars represent the standard errors (*n* = 3). Different lowercase letters indicate significant differences in antibiotic concentration among different soil layers (*p* < 0.05, Tukey HSD test); (**b**) the concentration of four main antibiotic subcategories, error bars represent the standard errors (*n* = 3); (**c**) the concentration of specific antibiotics. CL1-CL5 represent the 0–5 cm, 5–10 cm, 10–20 cm, 20–40 cm, 40–60 cm soil depths of long-term livestock wastewater-applied soils; CK1-CK5 represent the 0–5 cm, 5–10 cm, 10–20 cm, 20–40 cm, 40–60 cm soil depths of control soil. The gray lines in the left side and top are the clustering analysis for the specific antibiotics and the sampling sites, respectively.

The total antibiotic concentrations in different depths of the soil samples ranged from 0.02 to 1.21 μg/kg, with antibiotic concentrations in 5–10 cm significantly higher than in the other soil depths after long-term livestock wastewater application treatment, while only trace antibiotic concentrations were found in the control soil adjacent to the livestock wastewater treated block. After the Shapiro–Wilk test, the total antibiotic concentration of the soil samples conformed to a normal distribution. The one-way ANOVA result showed that the antibiotic concentration exhibited significant differences among different soil layers of CL (*p* > 0.05), while there was no significant difference among the different soil layers of CK (*p* < 0.05) (Figure 1a). There were 13 antibiotics, belonging to quinolones (QNs), TCs, SAs, and MLs, detected in both livestock wastewater treated and untreated soil (Figure 1b). The concentrations and species of antibiotics also had significant differences in different soil depths (*p* < 0.05) (Figure 1b,c). TCs are mainly distributed in the top 10 cm soil layer, especially in the 5–10 cm layer. The concentration of TCs ranged from 0.11 μg/kg to 0.31 μg/kg. QNs, SAs, and MLs were all detected in all of the soil depths, the concentration ranged from 0.01 μg/kg to 0.22 μg/kg. But the concentrations of QNs were significantly higher than the other two. In terms of the specific antibiotics, oxytetracycline (OTC) belonging to the TCs had the highest residual concentration (0.94 μg/kg), followed by enrofloxacin (ENR) belonging to QNs (0.14 μg/kg), clarithromycin (CLR) (0.07 μg/kg) and roxithromycin (ROX) (0.03 μg/kg) belonging to MLs, and sulfamethizole (SMT) belonging to the SAs (0.03 μg/kg). The application of livestock wastewater significantly increased the concentrations of residual antibiotics in the farmland.

### 3.2. The Bacterial Community Profiles of the Investigated Farmland

**Figure 2:**
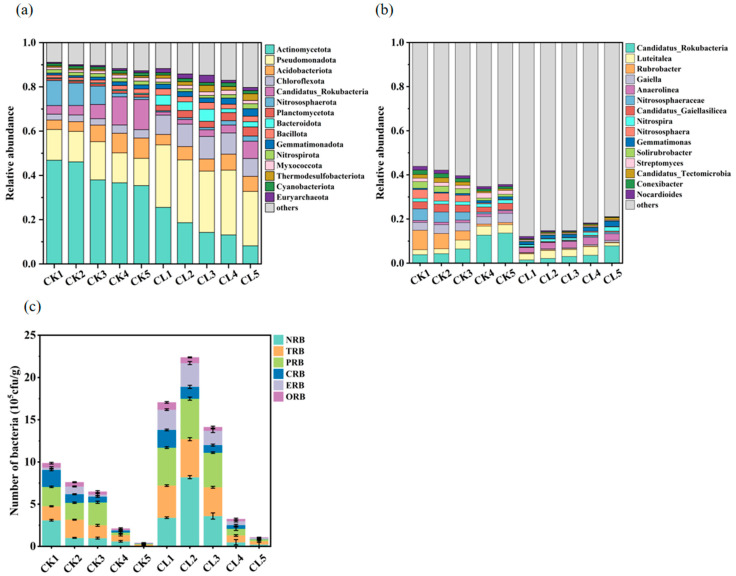
The bacterial community composition in different soil depths. (**a**) Relative abundance of top 15 bacteria at the phylum level. (**b**) Relative abundance of top 15 bacteria at the genus level. (**c**) The composition of ARB in soil under different depths. Error bars represent the standard errors (*n* = 3).

The relative abundance of bacteria in different soil depths was analyzed by metagenomic data annotation at the phylum level. Actinomycetota, Pseudomonadota, Acidobacteriota, and Chloroflexota were the four dominant phyla, which accounted for 47.6–67.7% of the total bacterial community (Figure 2a). The abundance of Pseudomonadota, Chloroflexota, and Bacteroidota phyla in CL soil was higher than the CK soil, which may be related to the long-term irrigation of the treated livestock wastewater. Actinomycetota was the most abundant bacterial phylum, which was more abundant in CK (35.4–46.9%) than that in CL (8.2–25.6%), and its proportions decreased significantly with the increase in soil depth. The abundance of the top 15 bacteria was significantly higher in CK soils than in CL at the genus level (Figure 2b). Moreover, the abundance of the top 15 genera gradually increased with the increase in soil depth. There was, however, one exception, the abundance of *Anaerolinea* sp. belonging to the Chloroflexota phylum was significantly higher in CL than in CK and evenly distributed in different soil depths.

Further screening of antibiotic-resistant bacteria using resistance plates revealed that there were 6 ARB, including norfloxacin-resistant bacteria (NRB), tetracycline-resistant bacteria (TRB), polymyxin-resistant bacteria (PRB), ciprofloxacin-resistant bacteria (CRB), oxytetracycline-resistant bacteria (ORB), and erythromycin-resistant bacteria (ERB) (Figure 2c). The viable ARB in all depths of the CL was higher than those in corresponding depths of the CK soil, which was mainly distributed in 0–5 cm, 5–10 cm, and 10–20 cm depths. The whole abundances of the ARB in the 0–5 cm, 5–10 cm, and 10–20 cm depths of the CL soil were 1.6–2.8 times higher than that of the CK. NRB, TRB, PRB, and ERB were the four main viable ARB.

**Figure A1:**
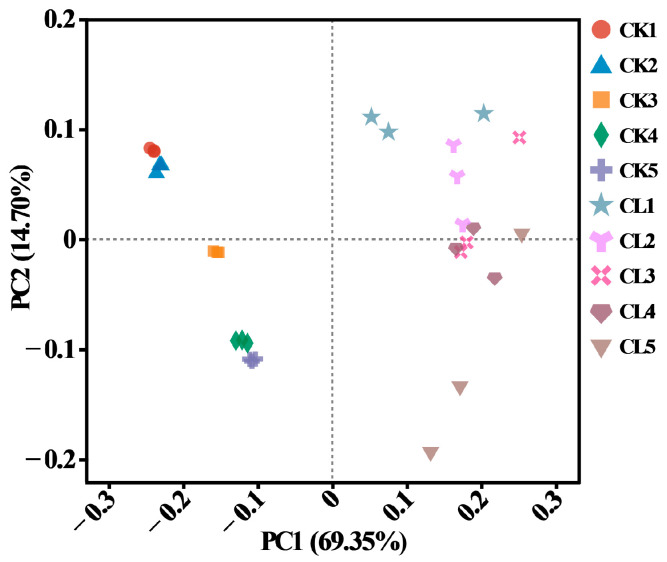
PCoA analysis of bacteria communities at the phylum level in different soil depths based on Bray–Curtis distances.

**Figure A2:**
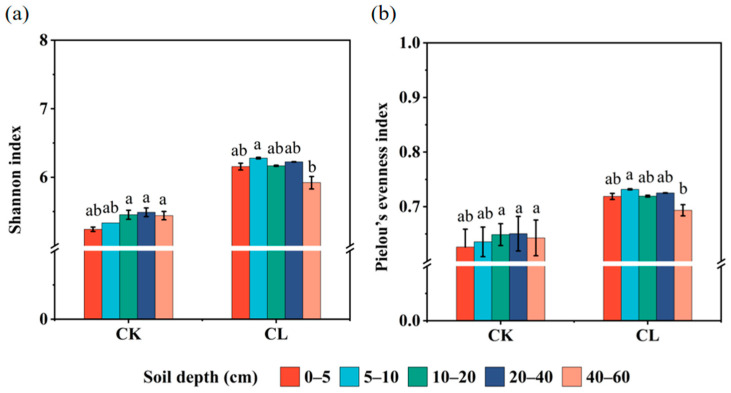
The α-diversity of the bacterial communities at genus level under different depths. (**a**) The Shannon index of bacterial communities. (**b**) The Pielou’s evenness index of the bacterial communities. Different lowercase letters indicate significant differences in α-diversity at different soil depths (*p* < 0.05, Tukey HSD test). Error bars represent the standard errors (*n* = 3).

The bacterial community structure had significant differences when comparing CK and CL at different soil depths using PCoA analysis based on the Bray–Curtis distance (Figure A1). Principal components 1 and 2 explain 69.35% and 14.70% of the variation in soil bacterial community structure, respectively. Shannon and Pielou’s evenness index were used to evaluate the alpha diversity and evenness of bacterial communities in soil samples. The alpha diversity analysis showed that the Shannon index of the CK was lower than that of the CL, and the highest species diversity of the bacteria community was found in the CL2 soil, which was significantly higher than that of the CL5 (*p* < 0.05) (Fig. A2). The Pielou’s evenness index showed a similar trend compared with the Shannon index. The application of livestock wastewater increased the diversity and evenness of the soil bacterial community.

**Figure 3:**
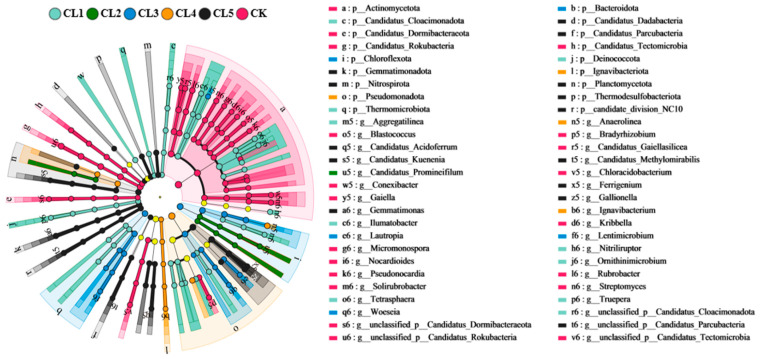
Dendrogram of the most differentially expressed bacterial taxa in different soil depths obtained by LEfSe analysis (LDA score > 3.5).

LEfSe analysis revealed that the dominant bacteria had significant differences among different soil depths (Figure 3). A total of 18 bacterial phyla were clustered into 6 groups (*p* < 0.01, LDA scores > 3.5). Actinomycetota was the dominant phylum in CK soil, followed by Candidatus_Dormibacteraeota and Candidatus_Rokubacteria. The main phyla had significant differences in different soil depths of the treated livestock wastewater-irrigated farmland. The main phyla were Candidatus_Cloacimonadota, Deinococcota, and Thermomicrobiota in 0–5 cm; Chloroflexota and Bacteroidota in 10–20 cm; Pseudomonadota and Ignavibacteriota in 20–40 cm; and Planctomycetota and Thermodesulfobacteriota in 40–60 cm. At the genera level, there were 16 predominant genera of bacteria in CK soil, among which 11 genera (such as *Rubrobacter* sp., *Gaiella* sp., and *Candidatus_Gaiellasilicea* sp.) belonged to Actinomycetota. But the high-abundant genera varied with soil depth in the CL soil. *Ilumtatobacter* sp., *Nitriliruptor* sp., *Aggregatilinea* sp., and *Tetrasphaera* sp., were the seven dominant genera in 0–5 cm of the treated livestock wastewater-irrigated farmland. *Candidatus_Promineifilum* sp. was the dominant bacteria in 5–10 cm, followed by *Lautropia* sp., *Lentimicrobium* sp., and *Woeseia* sp. in 10–20 cm; *Anaerolinea* sp. and *Ignavibacterium* sp. in 20–40 cm; and *Gemmatimonas* sp. and *Ferrigenium* sp. in 40–60 cm. The application of treated livestock wastewater had great influence on the diversity of soil bacterial communities except soil depth.

### 3.3. The Diversity and Abundance of the ARGs in the Investigated Farmland

**Figure 4:**
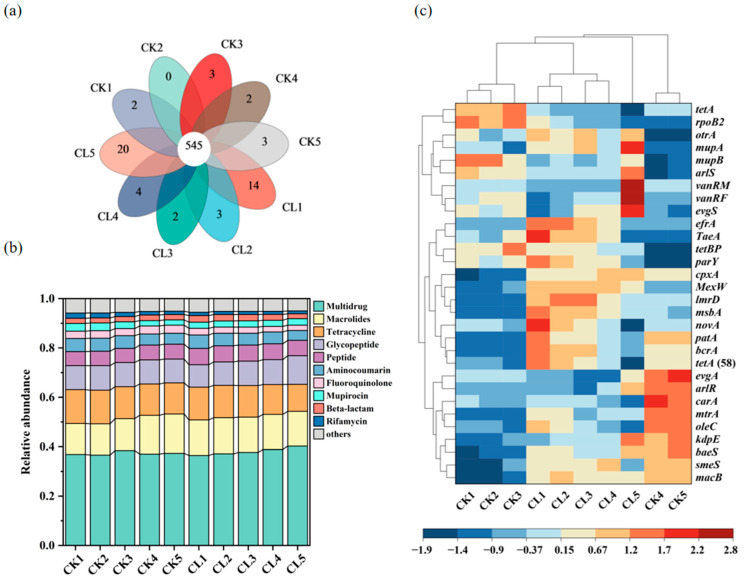
The number and abundance of ARGs in different soil depths. (**a**) The shared and unique ARGs presented by Wayne diagram. (**b**) Relative abundance of the top 10 ARGs types. (**c**) Relative abundance of the top 30 specific ARGs, the grey lines in left side and top are clustering analysis for the top 30 specific ARGs and the sampling sites, respectively.

**Figure 5:**
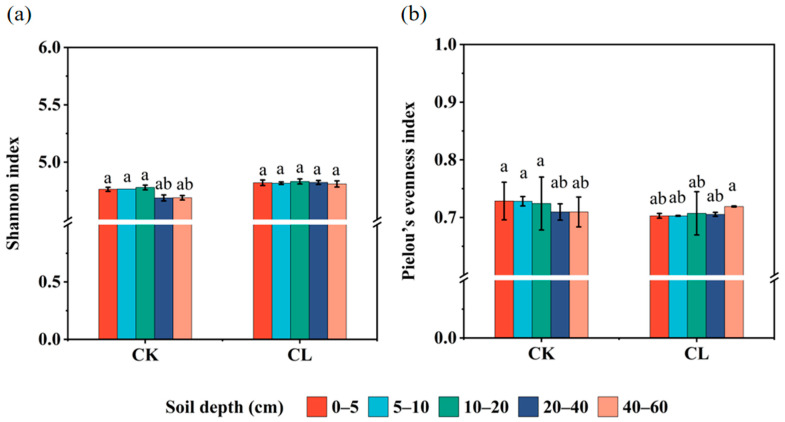
The α-diversity of the ARGs in different soil depths. (**a**) The Shannon index of the ARGs. (**b**) The Pielou’s evenness index of the ARGs. Different lowercase letters indicate significant differences of α-diversity among different soil depths (*p* < 0.05, Tukey HSD test). Error bars represent the standard errors (*n* = 3).

There were 545 common ARGs in all of the investigated soil samples, and the numbers of the specific ARGs in most of the soil depths of the CL were higher than the CK soil (Figure 4a). All of the identified ARGs were grouped into 21 antibiotic resistance categories, among which the ARGs with resistances to multidrug (36.46–40.24%), macrolide (12.55–15.92%), tetracycline (10.94–13.67%), glycopeptide (9.12–11.59%), and peptide (5.66–6.60%) were the most abundant ARGs in the investigated soil (Figure 4b). The relative abundances of the individual ARGs have significant differences among all soil depths (Figure 4c). Cluster analysis based on the Euclidean distance matrix revealed that the main ARGs in samples CL1, CL2, CL3, and CL4 were grouped together, and the ARGs in samples CK1, CK2, and CK3 were clustered into the other group. But the main types of ARGs in the deep soil layer had significant differences with the top layers of the soil, ARGs in sample CL5 and samples CK4 and CK5 were individually clustered into another two groups. The relative abundances of *efrA*, *TaeA*, *novA, patA*, *bcrA*, and *tetA* (58) in the CL1-CL4 samples were all higher than that of CK. *MupA*, *arlS*, *vanRM*, *vanRF*, and *evgS* were the high-abundance and unique ARGs in CL5 soil. *TetA*, *rpoB2*, and *mupB* were mainly distributed in the 0–20 cm layer of the CK soils, whereas *evgA*, *arlR*, *carA*, *mtrA*, *oleC*, *kdpE*, and *baeS* were predominantly distributed in the 20–60 cm depths of the CK soils. The α-diversity analysis showed that the Shannon indices of the CK were similar to those of the CL. The α-diversity data of all soil samples conformed to the normal distribution after the Shapiro–Wilk test. The one-way ANOVA result showed that the α-diversity of the ARGs had no significant differences among different soil depths (*p* > 0.05) (Figure 5a). The Pielou’s evenness indices of the 20–40 cm and 40–60 cm soil layers were lower than those of the 0–5 cm, 5–10 cm, and 10–20 cm of the CK (Figure 5b). The Pielou’s evenness index of the 40–60 cm layer was higher than the other depths of the CL soil, but the difference was not significant (*p* > 0.05).

### 3.4. Relationships Between the Main ARGs and the Bacteria

**Figure 6:**
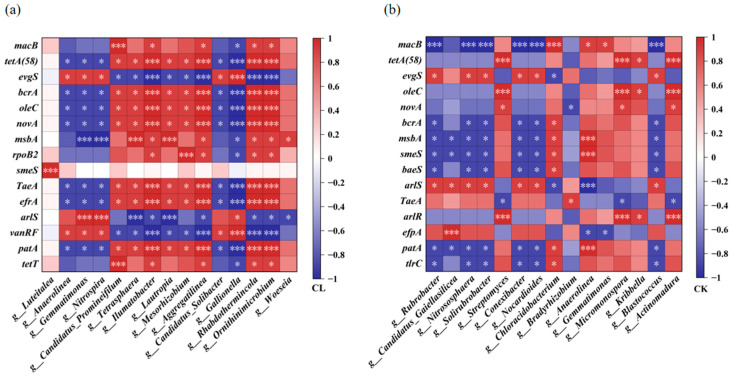
Heat map of the correlation between the top 15 bacteria genus and the top 15 ARGs in the soil samples of (**a**) CL and (**b**) CK based on Spearman analysis. (* *p* ≤ 0.05, *** *p* ≤ 0.001). Red/blue color in each cell indicate positive/negative correlations, and color gradient indicate the strength of correlations between the abundance of the specific bacteria genus and the ARG.

The top 15 bacterial genera and the top 15 ARGs were selected to analyze the correlation between ARGs and bacteria (Figure 6). The results showed that 12 of the 15 bacteria genera were significantly and positively correlated with the major ARGs in the CL soil samples, and 6 of the 15 bacteria genera were significantly and positively correlated with the major ARGs in the soil samples of the CK (Figure 6). *Ilumatobacter* sp., *Aggregatilinea* sp., *Rhabdothermincola* sp., and *Ornithinimicrobium* sp. had significant positive relationships with *tetA* (58), *bcrA*, *oleC*, *novA*, and several other resistance genes in CL soil (*p* < 0.001) (Figure 6a). *Streptomyces* sp., *Micromonospora* sp., and *Actinomadura* sp. had significant positive correlations with *tetA* (58)*, oleC*, and *arlR* in CK soil (*p* < 0.001). *Anaerolinea* sp. had significant and positive correlations with *msbA*, *smeS*, and *patA* in CK soil (Figure 6b). All of these potential host bacteria in the CK belonged to the phylum Actinobacteria.

### 3.5. Factors Influencing the Distribution of ARGs

**Figure 7:**
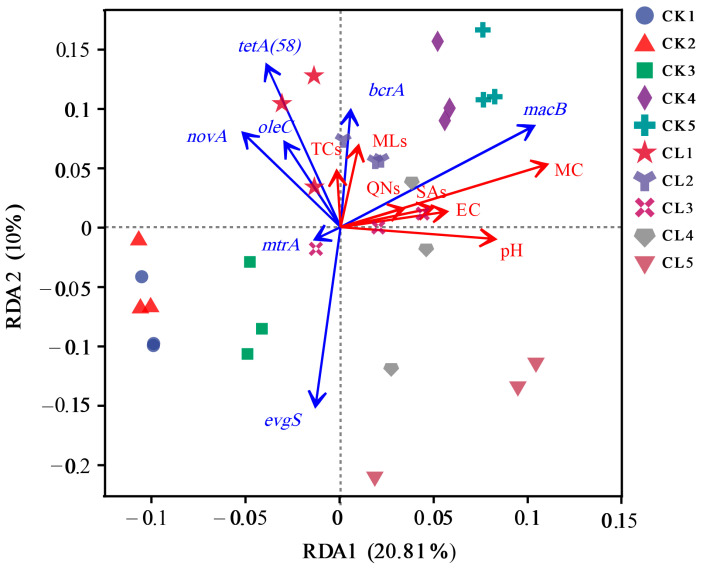
Redundancy analysis (RDA) of the correlation between the main environmental factors and the ARGs in different soil depths. The blue arrows represent the ARGs, and the red arrows represent the environmental factors.

Redundancy analysis (RDA) was used to investigate the effects of the main soil properties and antibiotic residues on ARGs (Figure 7). The results showed that RDA1 and RDA2 explained 20.81% and 10% of the distribution of ARGs, respectively. The soil sampling points were distinctly clustered into different groups. The main environmental factors, such as pH, EC, and MC were positively related to *macB* (*p* < 0.05) but negatively correlated with most of the other ARGs, such as *tetA* (58)*, oleC, mtrA, novA,* and *evgS*. The main types of antibiotics, such as TCs, QNs, SAs, and MLs, were all positively correlated with the main ARGs, such as *tetA* (58), *oleC, bcrA*, and *novA*, with the exception of *evgS*. Moreover, *oleC, bcrA*, and *novA* all belonged to multidrug resistance genes. The directions of the arrows of *tetA* (58), *oleC, bcrA*, and *novA* were all pointed or leaned toward the CL1, and the arrow of *macB* was leaned toward sample points CK4 and CK5, suggesting that these ARGs had higher abundances in corresponding sample points.

## 4. Discussion

### 4.1. The Influence of Long-Term Livestock Wastewater Application on the Residual Antibiotic Concentration of the Farmland Soil

Large amounts of antibiotics are used in high-density breeding processes to treat or prevent the occurrence of infectious diseases. However, most of the antibiotics cannot be fully utilized by the animals, and the residual antibiotics are excreted through feces and urine. Current wastewater treatment techniques focus on the removal of nitrogen and phosphorus, and the aerobic or anaerobic digestion process is inadequate to eliminate the residual antibiotics in the feces and urine. Excessive levels of residual antibiotics in livestock wastewater have been highlighted in previous studies. Sun et al. [28] reported that the total antibiotics concentration reached 121,502.74 ng/L in the raw swine wastewater of an intensive swine feedlot, the concentrations of the residual antibiotics were also at very high levels (lincomycin, 13,244.70 ng/L; doxorubicin, 7963.70 ng/L; and OXY, 4024.80 ng/L) after anaerobic treatment. The reuse of livestock wastewater is a common practice in agricultural and husbandry joint chains. But long-term application of livestock wastewater will lead to the accumulation of residual antibiotics in soil. In this study, all of the 13 investigated antibiotics were detected in the 20 soil samples from farmland soil, with a total concentration ranging from 0.02 to 1.21 ng/g, which was far lower than those in cultivated land of Zhangjiagang city (0.38–689.58 ng/g) [29] and the Huanghuaihai Plain region (1.62–575 ng/g) [30]. This may be due to the low frequency of the application of wastewater, with the residual antibiotics being degraded or absorbed into the soil. TCs, SAs, MLs, and QNs were the main types of antibiotic residues in our investigated soil, which also indicated that wastewater was the main source of antibiotics in farmland soil. But the residual antibiotics exhibited different distribution patterns across soil depths. TCs were only detected in the top 10 cm soil layer, while QNs, SAs, and MLs could be detected in the 0–60 cm soil layer. The benzene rings and lipid rings of TCs are adsorptive with the organic matters of soil particles, which resulted in a slow leaching and degradation rate [12]. But the *Kow* coefficients of QNs, SAs, and MLs are small, which led to their easily leaching into soil [31,32]. Zhang et al. [33] modelled and quantified antibiotics in groundwater and found a certain amount of antibiotics leached into groundwater, SAs account for more than 90% of antibiotics in groundwater, and the enhanced manure recycling practices were believed to be the main causes. In addition, previous studies showed that antibiotics in soil pore water were absorbed by plant roots, and part of it was transferred from roots to stems, leaves, and fruits [34]. Although the overall concentration of antibiotic residues in the investigated soil is not very high compared with the other previous studies [28,29,30], potential ecological risks still need to be considered, such as the continuous accumulation of hard-to-degrade antibiotics, food chain transmission, and the pollution of groundwater by antibiotics that are easy to leach [35].

### 4.2. The Influence of Long-Term Livestock Wastewater Application on the Distribution of ARB and ARGs in the Farmland Soil

The residual antibiotics in the soil exert selective pressure on the survival of surrounding microorganisms. Therefore, the application of incompletely treated livestock wastewater will inevitably facilitate the enrichment and dissemination of ARB and ARGs. In this study, the application of livestock wastewater significantly increased the number of ARB, especially TRB and ERB, which were significantly enriched in 0–20 cm surface soil. Accordingly, we found that the concentrations of TCs were 0.34–0.94 ng/g and only distributed in the 0–10 cm soil layer, and the concentration of erythromycin was the highest in the CL1 layer, which could reach 0.03 ng/g. This indicates that the ARB were significantly correlated with the concentration of the antibiotic residues. The inhibition effects of multiple antibiotics further incur the enrichment of ARGs and associated ARB. Previous studies showed that multidrug resistance genes and β-lactam resistance genes were significantly increased in the soils added with livestock manure [36,37]. In this study, long-term utilization of livestock wastewater also significantly increased the abundance of various ARGs in farmland soil, especially multidrug resistance genes. Furthermore, different ARGs may have the same host bacteria, and each particular ARG may also have many potential host bacteria [24], which leads to the frequent emergence of multidrug-resistant bacteria (MRB) containing different ARGs. In this study, *Ilumatobacter* sp., *Aggregatilinea* sp., *Rhabdothermincola* sp., and *Ornithinimicrobium* sp. carried the tetracycline resistance gene *tetA* (58), the multidrug resistance gene *bcrA, oleC, novA*, and several other resistance genes. ARGs may also migrate to groundwater and spread more widely around the environment.

Metagenomic sequencing was the main technology applied in the present research, and the top 15 bacteria and top 15 ARGs with high abundances were selected for further analysis. On this basis, the correlation between the bacteria and ARGs was obtained through correlation analysis. The relationships between specific bacteria and ARGs had been well elucidated. But it does not mean that the other bacteria and ARGs with low abundance were at low risk. Studies showed that some bacterial strain with low infective dose also had high potential to multiply in the human body [38]. But these harmful bacteria are hard to detect and quantify using current methods. In the CK soil, a certain abundance of ARGs were also detected, which may be due to the fact that some microorganisms in the soil secreted natural antibiotics, which exerted selective pressure on the surrounding microorganisms to obtain resistance. D’Costa et al. [39] identified ARGs encoding β-lactam, tetracycline, and glycopeptide antibiotics from 30,000-year-old frozen soil samples using metagenomic analysis. This study confirmed the existence of intrinsic ARGs in the original uncontaminated soil.

### 4.3. The Main Factor Influencing the Migration of ARGs in Soils

The distribution of ARGs is closely related to soil physicochemical properties. In this study, pH, EC, and MC had significant negative correlations with most of the ARGs, such as *tetA* (58)*, oleC, mtrA, novA,* and *evgS*. Alkaline environments, high conductivity, and water content had a negative effect on the proliferation of the ARGs in soil. Li et al. [40] found that raising pH facilitated the removal of pirlimycin and the degradation of *mefA* and *tet* (W) during the storage of manure slurries. The conformational landscape and electron densities of the antibiotics and the host bacteria of *mefA* and *tet* (W) were supposed to be sensitive to high pH, which in turn affected the abundance of corresponding ARGs. EC is an important index to measure soil salinity. Sun et al. [41] divided the sampled soil into different levels according to EC and found that salinity had low-dose promotion and high-dose inhibition effects on the abundance of ARGs, while medium-dose salinity was most conducive to bacterial growth and ARG propagation. Chen et al. [42] found that pH and EC were more crucial than high temperature in shaping the bacterial structure and the removal of the persistent ARGs (*sul1* and *sul2*), and regulation of these parameters was supposed to be effective for the elimination of these persistent ARGs. In this study, EC had a negative correlation with most of the ARGs except *macB*. We reasonably speculate that increasing soil EC within a normal range is helpful for the reduction of most of the ARGs. Furthermore, water content is also very important to the proliferation of ARGs. Wang et al. [43] found that paddy soil (rice) had higher accumulation of ARGs and MGEs than dryland (peanut), and the higher MC of paddy soil was thought to be one of the reasons. In this study, MC also had a negative correlation with most of the ARGs except *macB*. The reason might be that *macB* was different from other ARGs in host species, and they might experience different fates under different MC conditions. Consequently, the application of livestock wastewater can increase soil pH, EC, MC (Table A3), and antibiotic concentration, which strongly affects the composition and abundance of ARGs and the bacterial community structure in soil. The combined effects of these factors are still unclear, thereby posing an even greater threat to public health [44]. It is imperative to conduct a comprehensive study on the driving factors of ARG production and dissemination and to rationally regulate these factors associated with ARGs in future agricultural practices [45].

## 5. Conclusions

This study investigated the distribution of antibiotics, bacterial communities, and ARGs in different depths of the livestock wastewater-irrigated soils and explored the driving factors that influence the distribution of ARGs. It was emphasized that the concentration of antibiotics and the abundance and diversity of relative ARB and ARGs were significantly increased due to long-term irrigation of livestock wastewater. The distributions of antibiotics in different soil depths were obviously different. TCs were mainly found in the top 10 cm of the soil, while QNs, SAs, and MLs were detected in all 60 cm soil depths. The selection pressure of antibiotics led to the proliferation of ARB, especially tetracycline-resistant bacteria and erythromycin-resistant bacteria. In terms of the distribution/abundance of ARGs, *novA,* and *tetA* (58) were relatively higher in 0–10 cm surface soil, while *vanRM* and *vanRF* were mainly detected in deeper soil. Our findings reveal the influence of environmental factors on the spread of ARGs, especially soil pH, EC, and MC. Regulation of these parameters was supposed to be effective for the elimination of the ARGs. In the process of soil improvement, in order to stabilize the ecosystem, it should be carried out moderately to ensure that soil pH and EC are in the range suitable for crop growth. In the future, related measures based on these driving factors may have potential value in mitigating and controlling the proliferation of ARB and ARGs in agricultural soil.

## Data Availability

Data are contained within the article.

## Appendix A

**Table A1:** Collection parameters and detection limits for the analysis of target antibiotics.

Class	Antibiotic	Abbreviation	RT (min)	Precursor (m/z)	Adduct	Production (m/z)	CE (V)
SAs	Sulfadiazine	SD	1.44	215.1	[M + H] ^+^	156.0/92.2	10/22
	Sulphadimethoxine	SDM	2.875	311.00	[M + H] ^+^	156.05/92.05	−22/−35
	Sulfamethizole	SMT	2.058	271.00	[M + H] ^+^	156.00/108.00	−14/−23
	Sulfamethoxydiazine	SMD	2.699	281.10	[M + H] ^+^	156.10/108.10	−17/−26
	Sulfamethoxazole	SMZ	2.599	254.00	[M + H] ^+^	156.00/92.00	−16/−28
	Sulfaphenazolum	SPP	3.784	315.10	[M + H] ^+^	158.10/156.10	−28/−21
QNs	Ofloxacin	OFX	2.114	362.20	[M + H] ^+^	318.10/261.10	−18/−28
	Ciprofloxacin	CIP	2.393	332.20	[M + H] ^+^	288.10/314.10	−17/16
	Enrofloxacin	ENR	2.52	360.10	[M + H] ^+^	316.10/342.10	−20/−20
TCs	Oxytetracycline	OTC	2.258	461.20	[M + H] ^+^	425.95/443.10	−18/−14
MLs	Clarithromycin	CLR	6.695	425.30	[M + H] ^+^	126.10/377.30	−50/−20
	Erythromycin	ERY	6.455	734.30	[M + H] ^+^	158.15/576.35	−35/20
	Roxithromycin	ROX	6.72	837.50	[M + H] ^+^	158.15/769.40	−40/−25

Note: The superscript + denotes the single-charged cation formed after the molecule (M) combines with a proton.

**Table A2:** Elution gradients and ion source conditions for target antibiotics.

Time (min)	Elute A (%)	Elute B (%)	Flow Rate (mL/min)	Injection Volume (μL)	Spray Voltage (V)	ESI
0	85	15	0.4	10	4500	+
2	70	30				
5	60	40				
7	5	95				
8.5	5	95				
8.51	85	15				
12.5	stop					

**Table A3:** Physio-chemical properties of the sampled soils.

Sampling Site	Soil Layer	pH	EC (µS/cm)	MC (%)
CL	CL1 (0–5 cm)	9.84 ± 0.17 a	1461.3 ± 6.43 a	25.20 ± 1.91 b
	CL2 (5–10 cm)	9.66 ± 0.16 a	949.3 ± 13.28 b	23.59 ± 1.21 b
	CL3 (10–20 cm)	9.50 ± 0.21 a	872.8 ± 9.68b	33.16 ± 1.20 a
	CL4 (20–40 cm)	9.83 ± 0.12 a	679.3 ± 6.41 b	17.33 ± 0.95 b
	CL5 (40–60 cm)	9.95 ± 0.05 a	678.4 ± 10.12 b	20.40 ± 1.33 b
CK	CK1 (0–5 cm)	8.17 ± 0.012 a	69.6 ± 2.10 a	1.40 ± 0.29 d
	CK2 (5–10 cm)	8.15 ± 0.12 a	51.8 ± 2.65 a	4.19 ± 0.21 cd
	CK3 (10–20 cm)	8.21 ± 0.05 a	56.9 ± 2.20 a	7.11 ± 0.05 bc
	CK4 (20–40 cm)	8.41 ± 0.19 a	64.3 ± 3.39 a	10.61 ± 0.10ab
	CK5 (40–60 cm)	8.25 ± 0.21 a	54.7 ± 3.85 a	12.96 ± 0.19 a

Note: Data are presented as mean ± SE (*n* = 3). Different lowercase letters indicate significant differences among different soil depths (*p* < 0.05, Tukey HSD test).
